# Football (Soccer) as a Probable Cause of Long-Term Neurological Impairment and Neurodegeneration: A Narrative Review of the Debate

**DOI:** 10.7759/cureus.34279

**Published:** 2023-01-27

**Authors:** Daniele Ramsay, Alice Miller, Bibire Baykeens, Hamaas Hassan, Steve Gentleman

**Affiliations:** 1 School of Medicine, Imperial College London, London, GBR; 2 School of Medicine, Peninsula School of Medicine, Plymouth, GBR; 3 Brain Sciences, Imperial College London, London, GBR

**Keywords:** sports injury, amyotrophic lateral sclerosis, alzheimer’s disease, chronic traumatic encephalopathy, neurodegeneration, soccer, football

## Abstract

Football (soccer) is the most widely played sport across the globe. Due to some recent high-profile cases and epidemiological studies suggesting football can lead to neurodegeneration, scientific and public interest has been piqued. This has resulted in research into whether an association between football participation and neurodegeneration or neurological impairment is present. It has been theorised that a combination of repeated sub-concussive and concussive injuries, due to ball-heading and head collisions, may lead to neurodegeneration. However, evidence remains conflicting. Due to the popularity of the sport, and the serious conditions it has been linked to, it is important to determine whether repeated head impacts during football participation can play a causative role in neurodegenerative disease. To answer this question, a review of the current literature was carried out.

Epidemiological evidence showed a higher incidence of amyotrophic lateral sclerosis amongst amateur and professional footballers and that footballers in positions that involve less contact and heading, e.g., goalkeepers lead significantly longer lives. Additionally, imaging studies reach a similar conclusion, reporting changes in brain structure, blood flow, and inflammatory markers in footballers when compared to controls. However, studies looking at an association between heading frequency and cognition show a lack of consensus on whether a higher heading exposure results in reduced cognition. Similarly, in neuropathological studies, signs of chronic traumatic encephalopathy (CTE) have been found in some former players, with contrasting studies suggesting low levels of CTE-type pathology are found in the general population, regardless of exposure to head trauma.

The majority of studies suggest a link between football and neurodegenerative disease. However, the high prevalence of retrospective cohort and cross-sectional studies, often plagued by recall bias, undermine the conclusions drawn. Therefore, until larger prospective cohort studies are conducted, concrete conclusions cannot be made. However, caution can be exercised to limit head impacts.

## Introduction and background

Football (soccer) is the world’s most popular sport, with an estimated 250 million participants [1]. Following the evidence brought to light by epidemiological studies [2], growing interest has been expressed in understanding the association between football and increased rates of neurodegenerative disease in participants.

Neurodegeneration forms an overarching category that includes a range of conditions, such as Alzheimer’s disease (AD), Parkinson’s disease (PD), and amyotrophic lateral sclerosis (ALS). These diseases are caused by changes in neuronal structure, loss of function, and cell death in a progressive manner. The true nature of these diseases is still poorly understood, with pathological observations not always correlating with clinical symptoms. Chronic traumatic encephalopathy (CTE), for example, may present with cognitive impairment, increased suicidal ideation, or remain asymptomatic even with extensive pathology [3].

Many neurodegenerative conditions share mechanistic features at the cellular level. The common principal causes include protein dysfunction, neuroinflammation, and oxidative stress [4]. Some of the proteins involved may have a role in several diseases; one such example is hyperphosphorylated-tau, which has been implicated in both AD and CTE [5], where hyperphosphorylated-tau is found in neurons and astrocytes at the base of cortical sulci in a perivascular distribution [6].

The risk factors of neurodegenerative disease can vary, with recent studies beginning to unravel the interaction between genetic predisposition and environmental risk factors. One example is traumatic brain injury (TBI), which has been associated with an increased risk of conditions such as AD and PD [7-10]. Despite these more recent advances in understanding, ageing is still the greatest risk factor for neurodegenerative disease.

Understanding the changes that occur leading to neurodegeneration is often difficult as diagnoses are confirmed on neuropathological analysis. Therefore, other modalities must be employed in the form of cognitive and behavioural testing and imaging studies such as magnetic resonance imaging (MRI). In addition, particularly in the context of contact sports, determining the frequency and severity of a head impact often relies on self-reports, and these frequencies will often be recollected incorrectly. There is also a growing debate surrounding the definition of concussion and its value in describing TBI severity [11]. Despite this, the term is still commonly used and has been included for the purpose of this review.

In recent years, reports of CTE in former American football players [12,13] have prompted research into other contact sports, such as football, and whether they, too, are associated with neurodegenerative changes. Playing football involves a high degree of physical contact and, as a result, both subconcussive and concussive injuries can occur. While concussions usually produce noticeable symptoms, such as headache and dizziness [14], subconcussive injuries do not produce immediate neurological deficits [15]. However, a growing body of literature is linking factors such as repetitive heading with brain damage [16]. These studies provide a possible explanation for the increased rates of long-term neurodegeneration in footballers.

There remains much discourse on the subject, with studies finding evidence both for and against an association between football and long-term neurodegeneration. The topic has also received significant media coverage, partly due to news that Jeff Astle and other high-profile footballers died of CTE, which has led to much public concern [17]. This has even prompted the United Kingdom (UK) Football Association to publish guidelines on limiting heading in training [18].

Our principal aim was to examine the literature to determine if these concerns are justified by considering the evidence for and against there being an association between playing football and long-term neurological impairment and neurodegeneration. We have categorised our findings based on the following study types: epidemiological, cognition, pathological, and imaging.

## Review

Evidence for and against a correlation by study type

Research studies conducted across a range of academic disciplines have found evidence that both corroborate and dispute an association between repeated head impacts (RHIs) in football and long-term neurodegeneration. We explore the current evidence across these study types.

Epidemiological Studies

Epidemiological studies allow us to investigate the potential link between football as an exposure and neurodegenerative disease as an outcome over time. One particularly important research effort has been that of the Football’s Influence on Lifelong Health and Dementia Risk (FIELD) study [19-21,2], a collaborative effort investigating physical and mental health outcomes in former football players based in Scotland. Additionally, several other cohort studies across Europe have also examined this correlation.

Chiò et al. found a statistically significant link between a career lifespan of more than five years and ALS when studying 7,325 male Italian professional football players [22]. A follow-up study identified three further cases, particularly amongst midfielders, strengthening this correlation [23]. No ALS cases were found in a group of basketball players and road cyclists, suggesting that football is a specific risk factor for ALS. RHIs from heading and other risk factors, such as genetic predisposition, for example, mutations in the SOD1 and TDP-43 genes [24], and chemicals such as herbicides [25] are some explanations proposed by the authors. There are studies that corroborate the link between RHIs and increased neurodegenerative risk. Tierney and Higgins quantified the frequency of headers during 7,147 football games in six European leagues for three seasons by reviewing match footage and found that defenders consistently performed the greatest number of headers [26]. Interpreted with the results from a retrospective cohort study on former male professional Scottish footballers by Russell et al., which revealed that defenders had the highest risk of neurodegenerative disease [19], it could be argued that RHIs are a potentially significant risk factor. This would have to be further investigated with robust longitudinal studies before making definitive conclusions. An additional piece of supportive evidence came from the study performed by Russell et al., who found that the risk of neurodegenerative disease was lowest for goalkeepers [19]. Comparably, Śmigielski et al. observed that goalkeepers had a five-to-eight-year longer lifespan amongst international football players born before 1923 who played in the first three World Cups or in the 1946/1947 season of the main European leagues [27]. In general, it appears that the risk is high in footballers of all playing positions, as supported by Russell et al. [19] and Mackay et al. [2], whose study revealed a higher mortality rate from neurodegenerative disease amongst 7,676 former Scottish professional football players as part of the FIELD study; a higher frequency of prescribed dementia-related medications was also noted in this population.

In addition to a potentially higher risk of ALS amongst footballers, results from some studies also suggest an earlier onset. Pupillo et al. discovered 34 ALS cases, nearly double the expected number, amongst 23,586 male professional Italian players who were followed up from the age of 15 until either 2018 or their death [28]. The mean age at diagnosis was 20.2 years younger than that of the general population. Moreover, Gamez and Carmona found the mean age of ALS symptom onset to be 23.7 years younger [29]. However, these studies had several limitations, such as basing the results on incomplete medical records and having reduced statistical power from limited case numbers.

Ultimately, strong evidence can be obtained through cohort studies as temporal associations can be observed. Despite this, further longitudinal studies, including prospective matched-cohort studies, are needed before drawing definitive conclusions on the causality between football and neurodegenerative disease risk.

A summary of epidemiological studies investigating the association between participation in football and neurodegenerative disease is given in Table 1.

**Table 1:** A summary of epidemiological studies conducted investigating the association between participation in football and neurodegenerative disease. ALS: amyotrophic lateral sclerosis; SMR: standardized morbidity ratio; DOB: date of birth

Epidemiological studies					
Study	Study design	Method	Key Findings	Strengths	Limitations
Russell et al. (2021) [19]	Retrospective cohort	Population-based health record linkage in Scotland was used to evaluate risk in male former professional soccer players (n=7,676) and controls from the general population (n=23,028)	Football players had a 3.5 times greater risk of neurodegenerative disease compared to controls. Defenders had the highest risk and goalkeepers the lowest (similar to the general population). No difference in risk of neurodegeneration in footballers who played during an era that used heavier leather balls compared to use of leather and lighter synthetic balls.	Individuals in the two cohorts were matched by year of birth, sex, and area socioeconomic status.	Does not consider the length of participants’ non-professional career, age when started playing footballer, and whether other contact sports were played.
Mackay et al. (2019) [2]	Retrospective cohort	Mortality from neurodegenerative disease among former Scottish professional football players (n=7,676) was compared with that of the controls from the general population (n=23,028)	Mortality from neurodegenerative disease was higher among the former footballers (1.7% compared to 0.5% among controls) but was lower for mortality from other common diseases, e.g., ischemic heart disease, up to the age of 70, after which it was higher. Dementia-related medications were prescribed more frequently to former footballers than to controls.	A large sample size was used for both cohorts. The players and controls were matched by sex, age, and degree of social deprivation.	Not all former footballers identified from the master data sets of professional players could be matched to Community Health Index numbers to check the likelihood of full name and DOB belonging to the same person, therefore this may have affected the results.
Chiò et al. (2005) [22]	Retrospective cohort	ALS cases were identified in Italian male professional football players (n=7,325) from 1970–2001 and standardized morbidity ratios (SMRs) were then calculated for the players	A statistically significant link between football playing time (>5 years of playing) and ALS. Footballers had a higher SMR (6.5) than the general population (0.77).	The study population was defined according to very strict parameters, and cases that did not fit criteria were excluded.	No matched comparison group(s).
Chiò et al. (2009) [23]	Retrospective cohort	A follow-up of the Chiò et al. (2005) study over an extended five-year period. Cohorts of Italian professional basketball players (n=1973) and road cyclists (n=1701) were also included.	Three further ALS cases were found among the football players. No ALS cases were found among the basketball players and the road cyclists.	Provides further evidence supporting the original study, with two comparison groups.	Potential confounders, such as family history of ALS and smoking history, were not collected and adjusted for in the data analysis.
Tierney et al. (2021) [26]	Retrospective cohort	Quantified the frequency of heading during 7147 football games across six European football leagues for three seasons by reviewing match footage.	Defenders consistently performed the greatest number of headers.	Analysed heading frequency over a wide range of football games across Europe over three seasons.	Does not take training statistics into account.
Śmigielski et al. (2020) [27]	Retrospective cohort	Elite international football players born before 1923 who took part in the first three football World Cups (n=443) or played in the 1946/1947 season in the leading clubs of the main European leagues (n=280) were studied to determine if playing position affected lifespan	Goalkeepers had a 5-8-year longer lifespan compared to their colleagues playing in the field positions. There were no significant differences between the lifespan of defenders, midfielders, and forwards. The lifespan of footballers was distinctly higher than that of the male population in the second half of the 20th century (approximately 60 years globally and approximately 70 years in Europe).	Provides specific data on the effects of playing positions on mortality.	Confounders, such as risk factors for mortality (smoking, alcohol consumption etc.) and medical history were not analysed. The results are valid for elite football players born before 1923 who were at the peak of their professional careers more than 60 years ago.
Pupillo et al. (2020) [28]	Retrospective cohort	Demographic and career data was obtained for all professional Italian football players in the period 1950-2000 (n=23,586) Each player was retrospectively followed from when they were 15 until 2018 or death and any cases of ALS were noted.	Thirty-four ALS cases were identified, more than the 17.8 expected cases. The mean age at diagnosis (45 years) was lower compared to the general population (65.2 years).	Large sample size and long follow-up period.	Detailed medical records were only available for 18 cases. The remaining cases were confirmed using multiple independent sources. The age at diagnosis was not available in some cases and it was estimated using the mean disease duration.
Gamez et al. (2021) [29]	Retrospective cohort	Age and site of onset, survival time, history of trauma, playing position and time between retirement and first symptoms were investigated for footballer players in the Spanish league diagnosed between 2000 and 2020.	Seven ALS cases were identified. The mean age at onset was 41.5 years, 23.7 years younger than the general population. Two cases were goalkeepers, two defenders and three midfielders. Four cases had a history of trauma (two serious).	A search for ALS cases was carried out across multiple sources.	Though the authors state the study has the largest sample size of non-Italian league football professionals and semi-professionals for investigating ALS in footballers, the sample size is not stated. Four of the seven cases had a history of trauma, which may have contributed to their development of ALS.

Cognition Studies

Studies looking at the impact of football on cognition allow us to determine if football impacts an individual’s functionality. Research primarily looks at this in two populations, either those who are young and actively playing football or those who are older and retired from the sport. Although cognition does not always reflect the extent of neuropathology present, it can serve as a marker of neurodegeneration, particularly when there is a progressive effect on cognition.

Stewart et al. [30] and Levitch et al. [31] showed that active players with greater repetitive heading in the two weeks prior to cognitive testing being carried out had decreased psychomotor speed, memory, and attention. These same deficits are also seen acutely following concussion [30]. This suggests that, although sub-concussive, heading can induce similar cognitive deficits to concussion.

Interestingly, Levitch et al. also found that moderate levels of short-term heading (defined as occurring within the previous two weeks) improved attention levels [31]. Strauss et al. echoed this, finding footballers with no or low exposure to repetitive heading exhibited better attention, processing speed and memory compared to non-athletes [32]. However, once players pass a ‘threshold value’ in heading frequency, these benefits are no longer seen, with athletes with high heading frequency showing decreased cognition compared to other footballers [31].

Furthermore, studies have shown that active players with a greater history of long-term frequent heading (lasting more than 12 months) had lower cognitive testing scores in sections regarding attention, planning, verbal learning, and memory [31,33-35]. Levitch et al. theorised that this long-term reduction is due to the accumulation of damage induced by short-term heading, thus suggesting heading contributes to long-term neurodegeneration in footballers [31]. This conclusion is further supported by studies showing that amateur footballers performed worse in cognitive testing than controls [36,37].

Of note, participants in these studies were relatively young and all but one study used amateur footballers as opposed to professionals. This means that the individuals may not have had sufficient exposure to football-related head impacts to induce the levels of cognitive deficit and neurodegeneration that may predispose one to diseases such as CTE.

Koerte et al. [38] and Bruno et al. [39] addressed this by carrying out cognitive testing on retired professional footballers and found that these cognitive deficits persist following retirement. They found that retired footballers with a greater history of heading had reduced cognitive performance [39] and that footballers had worse memory than age-matched controls [38].

A limitation of many of these studies is that they depended on participants to report their own heading frequencies. This may have led to the collection of inaccurate data, especially considering that heading has been associated with worse memory. Future studies should use video recordings of games and training to corroborate data, thus reducing self-reporting bias. Additionally, confounding factors such as the force with which a ball was headed, rotational versus linear force experienced, participation in other contact sports, and level of football exposure during childhood and post-retirement were often not accounted for.

Cognition studies have been useful in identifying factors that may contribute to long-term neurodegeneration in footballers. They have also shown that the cognitive decline in retired professional footballers is more advanced than in the general population [38]. However, due to the cross-sectional nature of these studies, it is difficult to determine whether the cognitive decline seen is truly due to football and whether participation contributes to the development of long-term neurodegenerative diseases such as CTE. While there is evidence pointing to a dose-dependent relationship between heading frequency and neurodegeneration [30,39], studies that are longitudinal with larger sample sizes should be carried out. These should follow players throughout their careers and into retirement, helping establish the timeline and natural history of cognitive changes that occur following exposure to football. These should be followed by post-mortem pathological examination of participants.

Conversely, many studies investigating the cognitive effects of football have failed to observe any long-term impairments among players. Both Straume-Naesheim et al. [40] and Guskiewicz et al. [41] demonstrate that heading exposure or the number of unintentional RHIs, such as concussions, does not correlate with a worse neuropsychological performance on testing. The authors, therefore, conclude that playing football does not lead to cognitive impairment during a player’s career, meaning that the risk of long-term neurodegenerative outcomes is low. However, a limitation of both these studies was that there was no player follow-up and, once again, large cohort longitudinal studies are needed to confirm these findings. This was addressed by Kemp et al., who, in a longitudinal prospective study, showed that professional football players after a five-year follow-up displayed no changes in neurology, psychology, or structural brain imaging [42]. However, it should be noted that the sample population was relatively young and research into an older population is needed. Additionally, even though older studies had found an association between a history of unintentional RHIs and reduced cognition, more recent studies, such as those by Stewart et al. [30], Lipton et al. [33], and Bruno et al. [39], have not found this. These differing conclusions may be due to a change in the management of unintentional RHIs over time.

Furthermore, Vann Jones et al. demonstrate that retired football players, overall, have no greater risk of cognitive decline and dementia than the general population [43]. They also found that, in contradiction to other studies [19,27,29,38], player position and career length were not associated with an increased risk. As a result, the study suggests that the link between football and long-term neurodegenerative outcomes is lacking, and any cognitive impairment that is observed in players may be reversible by the time they have retired. However, the relevance of these findings is limited as the study had a small sample size and no control group.

Pathological Studies

Analysing the brain’s pathology post-mortem offers a unique opportunity to identify any insults that may have occurred. To understand more about the potentially pathological relationship between football and neurodegenerative disorders, former footballers' brains have been examined.

Several initial case reports, such as by Hales et al. [44] and Grinberg et al. [45], highlighted that AD may be incorrectly diagnosed in the place of CTE in patients with a significant football history. The authors suggested a different explanation for the findings was needed, with the chronic injurious effects of playing football as a potential theory. However, there were no comparison groups, and, due to the possible coexistence of AD and CTE pathology, the extent to which these both contribute to clinical symptoms required investigation. The coexistence of multiple pathologies was also found in a FIELD study conducted by Lee et al., in which seven former footballers with dementia were examined neuropathologically post-mortem [46]. A mixed presence of CTE and other pathologies was seen in five of the footballers, with two participants deemed to have sufficiently extensive CTE to explain their dementia. One limitation common to the previous pathology studies was the lack of prospective data collection. This, however, was undertaken by Ling et al., where clinical data and information about the participants’ playing careers, including rates of concussion, were collected [47]. Out of the 14 footballers analysed, six displayed abnormalities of the septum pellucidum, consistent with a history of chronic RHIs and four had pathologically confirmed CTE.

These studies allude to the potential for neurodegeneration due to football participation. The cross-sectional nature of neuropathological studies makes it difficult to identify the relationship between potential causes and effects. Yet again, this highlights the need for prospective cohort studies followed by post-mortem analyses.

On the other hand, a pathological study by Iverson et al. points towards the lack of an association [48]. In a post-mortem analysis of individuals without a known history of RHIs, they found that six out of eight individuals were positive for hyperphosphorylated-tau in neurons, astrocytes, and cell processes in the cortical sulci. Of the six, five met the diagnosis of CTE. Findings from this study are important in demonstrating that RHIs are not essential for the pathogenesis of CTE, and we can, therefore, speculate that the higher incidence of CTE in football players may be due to factors other than head trauma. A drawback of the study, however, is that it did not set a threshold of pathology for diagnosing CTE. It has been established that small levels of hyperphosphorylated-tau are common amongst the general population [49], therefore, unless a commonly agreed diagnostic threshold is used across studies, some individuals with small levels of pathology may have been misdiagnosed with CTE. This is further reflected by the presence of ageing-related tau astrogliopathy (ARTAG), which shares features seen in CTE, and the two pathologies may well coexist [50].

A summary of the pathological studies describing the incidence of neurodegenerative disease in professional football players is given in Table 2.

**Table 2:** A summary of the pathological studies describing the incidence of neurodegenerative disease in professional football players. CTE: chronic traumatic encephalopathy; AD: Alzheimer's disease; TDP-43: trans-activation response element DNA-binding protein 43

Pathological studies					
Study	Study design	Method	Key Findings	Strengths	Limitations
Hales et al. (2014) [44]	Cross-sectional	Neuropathological examination (n=1)	Late-stage CTE in ex-football player	Detailed neuropathological diagnosis and one of the initial football-CTE case reports	Small sample size and no detail about head impact frequency
Grinberg et al. (2016) [45]	Cross-sectional	Neuropathological examination (n=1)	Mixed pathology of CTE and AD	Adds to the evidence of football-related CTE	Small sample size and no detail about head impact frequency
Lee et al. (2019) [46]	Cross-sectional	Neuropathological examination, cohort (n=7)	Five showed CTE pathology but also extensive AD	Detailed neuropathological diagnoses and retrospectively collected head impact frequency	Head impact frequency information collected from next of kin, recall bias
Ling et al. (2017) [47]	Cross-sectional	Neuropathological examination of retired football players (n=6)	Four showed CTE pathology, six AD, TDP-43 in six, cerebral amyloid angiopathy in five, hippocampal sclerosis in two, dementia with Lewy bodies in one and one with corticobasal degeneration	Prospective data were collected on head impacts from the patients themselves and detailed clinical information was also collected	Although 14 participants were initially recruited only six were examined neuropathologically which may have resulted in selection bias, longer-term case-control studies needed
Iverson et al. (2019) [48]	Cross-sectional	Post-mortem analysis of immunostained brain tissue in men without a history of repetitive neurotrauma (n=8)	Six out of the eight cases had CTE pathology. Five of the six cases met the diagnosis for CTE suggesting that CTE pathology can be present in individuals without a history of repetitive neurotrauma	Neuropathologist was blind to age, personal history, and clinical history so bias was minimised	A pathological threshold was not set for diagnosing CTE Patient histories were collected from family surveys so there may have been some inaccuracies reported

Imaging Studies

One of the few ways of studying the brain and its structure in vivo is through imaging. Modalities such as MRI have been most useful in identifying changes in brain structure and volume. These have yielded interesting results concerning the health of current footballers’ brains. Therefore, to further investigate the potential neurodegenerative effects of football, imaging studies have been carried out looking at a range of factors which may predict neurodegeneration.

Four separate studies [32,33,38,51], including one large longitudinal study conducted by Strauss et al. [32], used MRI to determine the impact of RHIs on white matter tracts, cortical thickness, and their relationship to cognition. They revealed that a high heading frequency was associated with poor white matter integrity and a reduction in bilateral parieto-occipital thickness, and this correlated with worse memory performance when compared to activity-matched controls. Another study [52] using magnetic resonance spectroscopy scanned 11 footballers with no history of head trauma and 14 age- and activity-matched non-contact sports controls. This study demonstrated a significant increase in neuroinflammatory markers, such as choline, myoinositol, and glutathione, in footballers, the latter two markers correlating with estimated lifetime headers. In addition to the neuroinflammatory role which may lead to the neurodegenerative effects seen in footballers, impaired cerebral blood flow regulation may also be implicated, as was demonstrated by a study conducted by Marley et al. [36]. The 16 amateur football players showed an equal baseline cerebral blood flow to age- and activity-matched controls. However, when challenged with hypo- and hypercapnia, the control group responded more effectively, as determined with the use of transcranial doppler ultrasound. To add to this, the footballers scored more poorly on cognitive testing, thus providing evidence that the sport contributes to neurodegeneration. Robust imaging studies have been conducted in athletes from different sports, for example in rugby players [53], and efforts should be made to adapt these methods for studying neurological outcomes in footballer players.

A summary of the cognition and imaging studies investigating the structural and neuropsychological correlates of football participation is given in Table 3.

**Table 3:** A summary of the cognition and imaging studies investigating the structural and neuropsychological correlates of football participation. ADHD: Attention deficit hyperactivity disorder; APOE: apolipoprotein E; CBF: cerebral blood flow

Cognition and Imaging studies					
Study	Study design	Method	Key Findings	Strengths	Limitations
Stewart et al. (2018) [30]	Cross-sectional	Adult amateur footballers underwent neuropsychological testing and were asked to report on heading level and incidents of unintentional head injury in the two weeks prior (n=308).	Higher levels of heading were associated with poorer psychomotor speed, attention and working memory. Unintentional head impact had no effect on neuropsychological performance.	Shows ‘dose-dependent’ relationship between heading frequency and performance on cognitive tasks, thus strengthening evidence they have a causal relationship.	Use of amateur footballers; Does not consider factors such as ball speed or force ball headed with; Did not control for participants playing other contact sports; Self-reported heading frequency
Levitch et al. (2018) [31]	Cross-sectional	Adult amateur footballers underwent neuropsychological testing and reported on frequency of heading in the prior 12 months and two weeks (n=311).	High levels of heading in the prior two weeks were associated with reduced psychomotor speed. High levels of heading in the prior 12 months were associated with poorer verbal learning, and verbal memory. Improved attention was associated with moderate levels of heading. Concussion history had no impact on neuropsychological performance.	Measure of cognition following both long- and short-term exposure to heading	Use of amateur footballers; Cross-sectional; Did not exclude individuals with ADHD; High proportion of participants had English as a second language, possibly impacting performance on cognitive testing; Self-reported heading frequency
Strauss et al. (2021) [32]	Cross-sectional	Adult amateur footballers (n=246), non-collision sport athlete controls (n=72) and non-athlete controls (n=110) underwent cognitive testing, diffusor tensor imaging, and were asked to estimate heading frequency.	Athletes with no or lower exposure to repetitive heading exhibited both a higher degree of white matter anisotropy and better performance on tasks of attention, processing speed, verbal memory, and working memory compared to non-athletes. Footballers with the highest exposure to repetitive head impacts did not differ significantly from healthy, non-athletes on either micro-structural features or cognitive performance.	Exclusion criteria included history of neurological disorders and history of head trauma. Use of controls. Assessment of confounding factors	Use of adult amateurs; Cross-sectional study design; Footballers playing other collision sports not excluded for
Lipton et al. (2013) [33]	Cross-sectional	Adult amateur footballers underwent cognitive testing, diffusor-tensor imaging and reported on heading frequency in prior 12 months and lifetime concussion incidence (n=37).	High heading frequency was associated with a reduction in white matter integrity and memory performance. No difference in fractional anisotropy or cognition was seen when comparing concussion frequency.	Combines cognitive testing with imaging	Use of amateur footballers; Cross-sectional; Small sample size; Does not consider factors such as ball speed or force ball headed with
Matser et al. (2010) [34]	Cross-sectional	Active male premier league professional footballers underwent neuropsychological testing and reported on heading and concussion exposure in the prior season (n=84).	A greater number of headers was associated with decreased verbal memory, visual memory and attention. A greater number of concussions was related to decreased attention and visuoperceptual processing.	Use of professional footballers Confounders thoroughly accounted for	Heading and concussion frequency obtained through interview leading to bias; Small sample size; Cross-sectional
Hunter et al. (2020) [35]	Cross-sectional	Amateur footballers underwent neuropsychological testing, genotyping and reported their heading frequency in the prior 12 months (n=352).	High levels of heading were associated with worse verbal memory performance. Found an association between APOE ε4 allele and performance, suggesting that APOE ε4 allele is a risk factor for worse memory performance associated with higher heading exposure.	Combined genotyping with cognitive testing	Use of amateur footballers; Self-reported heading frequency given subject to recall bias; The highest heading exposure subgroup had fewer years of education, which may explain the lower test scores.
Marley et al. (2021) [36]	Cross-sectional	Amateur football players (n=16) and controls (n=18) underwent cognitive testing and cerebral blood flow investigation	Basal CBF was equal across the two groups, but when challenged with hypercapnic or hypocapnic conditions, controls responded more effectively. Cognitive testing revealed worse scores in the football group.	Combining imaging and cognitive testing; Comparison to controls	Use of amateur footballers; Cross-sectional; Small sample size
Matser et al. (1998) [37]	Cross-sectional	Amateur footballers (n=33) and controls (n=27) underwent neuropsychological testing and were asked about concussion history.	Footballers displayed impairment in both memory and planning testing when compared to controls. A greater number of concussions in footballers was associated with worse cognition.	Comparison to controls Through assessment for confounding factors that may affect cognition	Use of amateur footballers; Cross-sectional design; Small sample size
Koerte et al. (2016) [38]	Cross-sectional	Former professional footballers (n=15) underwent an MRI, cognitive/behavioural testing and were asked about heading frequency. They were compared to controls (n=15).	Revealed a decrease in cortical density in the footballer group which was determined not to be as a result of normal ageing. A negative correlation between heading frequency and cortical thickness was confirmed with the greatest lifetime heading frequency having the thinnest cortex. The single goalkeeper in this study, a position with very limited heading involved, had the greatest age-adjusted cortical thickness. The neurocognitive and behavioural tests revealed a significant difference in the ability to recall after prolonged periods of time.	Comparison with controls Use of former professional footballers	Small sample size; Lack of objective measures for quantifying headers
Bruno et al. (2021) [39]	Cross-sectional	Former professional footballers were asked about their career including history of heading and head injury and asked to complete cognitive testing (n=60).	Greater estimated career heading frequency was associated with reduced cognition; No association between career head injury history and cognition was found. Sixteen out of the 60 study participants scored below the age-adjusted questionnaire scores suggesting the presence of mild cognitive impairment.	Use of former professional footballers	Self-reported questionnaire used, prone to recall bias; Due to enrolment technique, subject to selection bias.
Koerte et al. (2012) [51]	Cross-sectional	Professional football players (n=12) and matched swimmers (n=8) underwent high-resolution diffusion tensor imaging	Decrease in white matter tract integrity in footballers	Good discussion of the results and the authors did not overstate their findings	Cross-sectional study and self-reported head impact data
Koerte et al. (2015) [52]	Cross-sectional	Magnetic resonance spectroscopy and neurocognitive evaluation were carried out on former professional football players (n=11) and age and gender-matched non-contact athletes (n=14)	Significant increases in choline, myoinositol and glutathione in footballers, these are markers associated with injury, inflammation and glial activation	Range of biomarkers used as well as cognitive testing alongside the main imaging	The raised markers do not differentiate between short- or long-term injury, cross-sectional study, and self-reported head impact data
Straume-Naesheim et al. (2005) [40]	Cross-sectional	Professional Norwegian football players underwent baseline neuropsychological tests and completed a questionnaire on heading exposure and concussion history (n=271)	No correlation was found between neuropsychological test performance and heading exposure or concussion history	High response rate and sample size	Data is self-reported so is subject to recall bias; No player follow-up; The sample population was relatively young
Guskiewicz et al. (2002) [41]	Cross-sectional	Collegiate football athletes, non-football athletes, and non-athlete controls completed a questionnaire on previous concussions, heading exposure and Scholastic Aptitude Test results. They also performed baseline neuropsychological tests (n=242)	No changes in neurocognitive function or scholastic aptitude were observed in football athletes when compared to non-football athletes or nonathlete controls. There was no significant association between a history of football-related concussion and neurocognitive performance or scholastic aptitude.	Large sample size. Inclusion of controls	Data is self-reported so is subject to recall bias; No player follow-up; The sample population was relatively young
Kemp et al. (2016) [42]	Prospective cohort	A longitudinal prospective study was carried out on footballers and controls. Medical examination, MRI imaging, and neuropsychological testing were carried out at baseline and after a five-year follow-up (n=65)	No significant changes were observed in neurology, structural brain imaging or neuropsychological testing amongst professional footballers when compared to controls over a five-year period	Longitudinal prospective study design Inclusion of controls	The sample population was relatively young; Some participants were lost to follow-up
Vann Jones et al. (2014) [43]	Cross-sectional	Former football players completed the Test Your Memory questionnaire (n=92)	Former football players did not have a greater risk of cognitive decline and dementia than the general population. Player position or career length was not associated with a greater risk either	Study carried out in an older cohort	Small sample size; Potential for selection bias; Data is self-reported so is subject to recall bias

Summary

Evidence from some pathological studies appears to suggest that football may be inflicting injurious effects on the brain, which may be leading to the development of neurodegenerative conditions, particularly CTE. The findings by Hales et al. [44] and Grinberg et al. [45] highlight the potential for an AD misdiagnosis in former footballers displaying possible symptoms of CTE. Lee et al. [46] and Ling et al. [47] also found the presence of CTE mixed with other neurodegenerative pathologies, such as AD. Given that CTE is largely associated with RHIs, these findings raise the question of whether physical trauma encountered during football, including headers and head-to-player impacts, plays a significant role in the manifestation of clinically diagnosed neurodegeneration. However, not all pathological studies corroborate this. Iverson et al. found individuals without a known history of RHIs with pathological findings associated with CTE, with most meeting the diagnostic criteria for CTE [48]. This suggests that the development of the pathological signs of CTE could be due to reasons other than RHIs, challenging football as a potential risk factor. However, small levels of hyperphosphorylated-tau are common amongst the general population [49] in the form of ARTAG [54]. Therefore, the results may have been skewed by diagnosing CTE based on a baseline threshold that is not clinically significant. Nevertheless, pathological studies suggesting a potential link between football and CTE have several limitations, such as their cross-sectional nature and small sample sizes, which means that a definitive conclusion cannot be confidently drawn and further post-mortem analyses following prospective cohort studies are needed. The combination of a confident assessment of RHI burden during life; accounting for lifestyle and genetic factors; and detailed neuropathological analyses.

Studies looking at the cognitive effects of football have found that heading is associated with reduced cognition among players. Studies by Koerte et al. [38] and Bruno et al. [39] have especially highlighted that reduced cognition due to football can persist until retirement, which may be indicative of long-term neurodegenerative changes. However, Levitch et al. [31] and Strauss et al. [32] found that low to moderate levels of heading may lead to improved cognitive abilities. Additionally, some studies have refuted the association between heading and cognitive decline entirely. In a cohort of retired football players, Vann Jones et al. demonstrated that long-term football participation did not cause an increased risk of cognitive decline or dementia; although, as previously mentioned, the relevance of these findings is limited because of the small sample size and lack of a control group [43]. Overall, whilst there is little evidence to refute an association between heading in football and reduced cognition, the evidence in favour of an association is insufficient. This is primarily due to the limitations of the study methodologies. Regarding unintentional RHIs, recent studies by Stewart et al. [30], Lipton et al. [33], and Bruno et al. [39] suggest that an association with cognitive decline does not exist.

Most imaging studies agree that playing football can lead to neurodegenerative changes in some individuals. This includes changes such as poor white matter integrity, reduced cortical thickness, increased neuroinflammatory markers, and impaired cerebral blood flow. From the studies included in this review, only Kemp et al. failed to detect any neurodegenerative changes through imaging, but this may be attributed to the sample population being young [42]. It is also impossible to deny the beneficial health effects of playing football, with evidence showing that the sport improves overall fitness [55] and, through moderate heading, cognitive ability [31].

## Conclusions

Overall, the literature suggests that a career in professional football is a possible risk factor for neurodegenerative disease. However, there may be other factors at play, such as genetic predisposition and environmental exposures. Nevertheless, there appears to be a paucity of studies providing counter evidence. Until more concrete evidence is found, we recommend that, rather than banning heading altogether, caution should be taken by minimising the amount of heading outside of matches. For example, introducing limitations of heading during training could provide a significant reduction in the RHI burden. We also recommend that leagues educate players about the possible neurological consequences of playing football. This will allow players to make informed decisions about the level of risk to which they expose themselves.

While most studies argue in favour of a correlation between football and long-term neurodegenerative disease, the evidence is insufficient to establish a causal relationship. This is mainly due to the methodological limitations of current studies. Additionally, we aimed to investigate the correlation between football and long-term neurodegenerative diseases, such as AD, PD, ALS, and CTE. However, most of the literature was focused on the link between football and ALS or CTE. This suggests a disproportionate focus on these disorders and highlights a need for further studies investigating a link with other neurodegenerative conditions, such as PD. Future work should focus on forming prospective, population-matched studies incorporating serum or imaging biomarkers for TBI and RHI. In addition, considerable effort should be directed towards the untangling of the clinicopathological presentations of CTE and other neurodegenerative conditions. This would greatly improve current research and provide considerable insight when developing future RHI management guidelines.
